# System-Based Approaches to Delineate the Antiviral Innate Immune Landscape

**DOI:** 10.3390/v12101196

**Published:** 2020-10-21

**Authors:** Karsten Krey, Aleksandra W. Babnis, Andreas Pichlmair

**Affiliations:** 1 School of Medicine, Institute of Virology, Technical University of Munich, 81675 Munich, Germany; karsten.krey@tum.de (K.K.); aleksandra.babnis@tum.de (A.W.B.); 2 German Center for Infection Research (DZIF), Munich Partner Site, 80538 Munich, Germany

**Keywords:** innate immunity, virus–host interactions, interferon-stimulated genes, genetic screens, gene regulation, knockout, overexpression

## Abstract

Viruses pose substantial challenges for society, economy, healthcare systems, and research. Their distinctive pathologies are based on specific interactions with cellular factors. In order to develop new antiviral treatments, it is of central importance to understand how viruses interact with their host and how infected cells react to the virus on a molecular level. Invading viruses are commonly sensed by components of the innate immune system, which is composed of a highly effective yet complex network of proteins that, in most cases, mediate efficient virus inhibition. Central to this process is the activity of interferons and other cytokines that coordinate the antiviral response. So far, numerous methods have been used to identify how viruses interact with cellular processes and revealed that the innate immune response is highly complex and involves interferon-stimulated genes and their binding partners as functional factors. Novel approaches and careful experimental design, combined with large-scale, high-throughput methods and cutting-edge analysis pipelines, have to be utilized to delineate the antiviral innate immune landscape at a global level. In this review, we describe different currently used screening approaches, how they contributed to our knowledge on virus–host interactions, and essential considerations that have to be taken into account when planning such experiments.

## 1. Introduction

Over millions of years, the innate immune system evolved into an effective defense barrier against pathogens. Harnessing the power of the immune system and unleashing its activity to fight pathogens at a broad level holds the promise to treat infectious diseases, as well as autoimmune diseases and cancers. However, the benefits of innate immune activation are accompanied by the risk of overshooting immune reactions, which may lead to tissue damage and autoimmunity. Further details of the functions of the innate immune system, particularly its complex regulatory network and cell-specific effects, need to be understood comprehensively. This complexity requires adequate approaches with respect to the characteristics of the tested pathogen and the analysis methods.

Here, we review experimental approaches to study the innate immune system on a systems level, in particular the benefits and limitations of current screening methods. The nature of the chosen screening approach used—Gain-of-function screens, loss of function screens, or physical interaction screens—Tremendously influences the conclusion that can be drawn from these experiments. Similarly, the readout of the screen has to be suitable for the scientific question that is asked. Moreover, since screening approaches mostly apply simplistic model systems to obtain high-throughput data, functional validation in more sophisticated systems, such as primary cells or living animals, is often necessary. Which parameters should be considered to identify candidates from omics screens? What are the benefits and pitfalls of the different screening approaches in the context of screens dissecting the innate immune system?

In the following, we describe methodological aspects of different screening platforms, which can be utilized to study the innate immune landscape on a systems level. We outline their advantages and limitations and discuss necessary considerations.

### 1.1. The Intracellular Antiviral Responses

**Figure 1:**
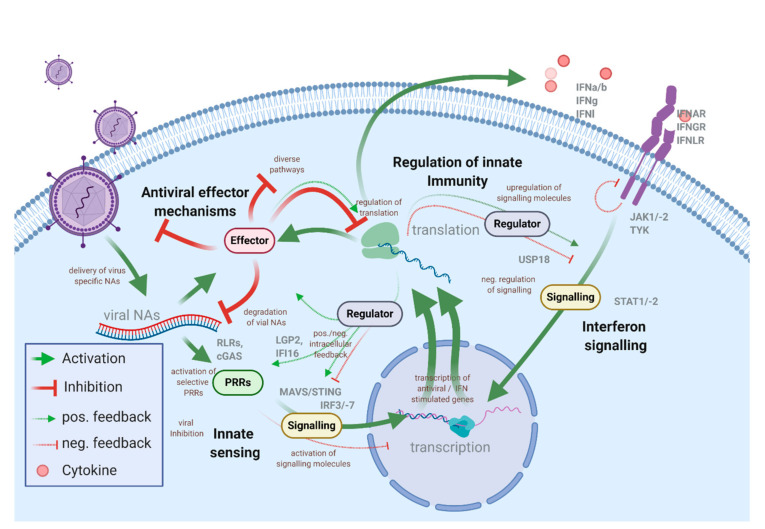
The antiviral innate immune response is orchestrated by signaling cascades and the synergistic activity of numerous factors that are converging in a complex regulatory network. Pathogen-associated molecular patterns such as viral nucleic acids (viral NAs) are sensed by cytoplasmic pattern recognition receptors (PRRs), which in turn, activate signaling cascades of the innate immune system. This leads to the expression of regulatory function (Regulator) and proteins with antiviral effector proteins (Effector), which is controlled on transcriptional and translational level. Paracrine and autocrine interferon (IFN) signaling synergistically modulate these signaling cascades. The activity of numerous contributing factors puts the cell into an antiviral state, inhibiting virus replication at multiple stages of the viral life cycle. To avoid overshooting immune reactions and adverse effects on cellular fitness, negative regulators eventually shut down the IFN response.

Viruses are vehicles that transport genetic information, which is amplified in the host cells. Even though the virion structure, infection, and replication strategies are very diverse, the unifying feature—delivery of viral nucleic acids and the requirement of the host machinery to produce progeny virus particles—is similar for all viral pathogens. The intracellular antiviral immune system is tailored to prevent virus spread and bridge the time until an adaptive immune response can be mounted. However, while some antiviral factors are constitutively present in cells and provide a steady level of intrinsic immunity, most of the proteins and pathways involved in the innate antiviral defense need to be induced. In the course of most virus infections, infected and non-infected bystander cells mount an antiviral response for a limited timeframe, controlled by a set of positive and negative feedback loops. This, however, costs cellular resources [1,2]. The activation of the innate immune system is under the control of pattern recognition receptors (PRRs) that sense invading pathogens through pathogen-associated molecular patterns (PAMPs), such as viral nucleic acids, proteins, or lipids [3,4]. The presence of viral PAMPs in the cell usually results in the synthesis of antiviral and pro-inflammatory cytokines, especially interferons (IFNs) (Figure 1).

Today the principal activation framework and key signaling mechanisms involved in these processes are known. However, more recent data, often obtained through screening approaches, indicate that regulation of the signaling cascades are surprisingly complex and involve sophisticated regulatory mechanisms and additional layers of control, which are only partially understood. Notably, it appears that this complexity is necessary to prevent detrimental effects related to innate immune activation [5,6,7].

A hallmark of innate immune activation is the transcriptional upregulation and secretion of antiviral (IFN-type) and pro-inflammatory cytokines. IFNs are classified in type I (13 IFN-α subtypes, IFN-β, IFN-ω, IFN-ε, and IFN-κ), type II (IFN-γ), and type III (4 subtypes of IFN-λ) [8]. Their classification is based on their ability to bind to distinct heterodimeric receptors (IFNAR1/2 for type I IFN; IFNGR1/2 for type II IFNs; IFNLR1/IL10Rβ for type III IFNs). The main difference between these receptors appears to be their expression profile among different cell types and their signaling strength. While IFNAR is widely expressed on most cell types, IFNLR is absent on lymphoid cells (i.e., leukocytes). Downstream signaling of IFNAR and IFNLR depends on kinases (JAK1, -2; TYK) that phosphorylate signal transducer and activation of transcription (STAT) 1 and 2 proteins. STAT1 and STAT2, together with IRF9, form a transcription complex (ISGF3) that binds to IFN-stimulated response elements (ISRE) of target genes that are therefore referred to as IFN-stimulated genes (ISGs) [9,10]. Type I and type III IFNs share their downstream signaling cascade and therefore induce a similar set of ISGs, however, with differences in potency and kinetics [11,12]. The composition of the ISG pattern is affected by additional regulators that influence the assembly of the transcription complex and involve epigenetic regulators and noncoding RNAs that contribute to the observable cell-type-specific differences [13,14,15].

The induction of ISGs is a common feature of the intracellular antiviral response in vertebrates. A global comparison of the IFN response among ten different vertebrate species led to the identification of a subset of 62 core vertebrate ISGs [16]. This cross-species conservation suggests a strong positive selection of these factors as crucial parts of the innate immunity. In humans, hundreds of genes are induced by IFNs. However, their function is only partially understood, often remains elusive, and is context-related, i.e., only active against certain pathogens or in specific tissues. Functionally, ISGs can be divided based on their mechanism of action. Some ISGs directly target viral proteins or nucleic acids, thus inhibiting viral entry, transcription, translation, genome replication, assembly, or budding (e.g., interferon induced proteins with tetratricopeptide repeats (IFIT proteins), MX proteins). Other ISGs act indirectly by affecting pathways and host factors required for virus replication (e.g., during PKR regulating translation, TDRD7 inhibits autophagy to suppress paramyxovirus replication [17]). Another group regulates the sensing of pathogens and modulation of downstream signaling (e.g., PRRs, IRF3, IRF7, and TRIM5). Many ISGs have numerous antiviral and regulatory functions and are involved in multiple processes. For example, tetherin that impairs viral budding also regulates NF-κB or RIG-I—which function as PRRs—and can displace viral proteins from viral RNA by its dsRNA binding domain [18,19,20]. Many unidentified functions and diverse modes of action of individual ISGs make the IFN-induced antiviral responses a complex topic that still needs to be understood more comprehensively [21].

Activation of PRRs and the expression of some ISGs significantly affect growth and viability, underlining the importance of tight regulation of the innate immune system. Consequently, the expression of cellular antiviral immunity effector proteins is well-controlled through positive and negative feedback loops (Figure 1). Well-known examples of ISGs that serve as regulators of the innate immune response are cofactors of PRRs that contribute to the activation of the innate immune system. IFN-induced LGP2 and IFI16 appear to be regulators of MDA5 and cGAS responses, respectively [22,23,24,25]. The group of proteins belonging to the suppressor of cytokine signaling (SOCS) family are negative feedback regulators of cytokine signaling, including IFNs, that act by blocking and inducing ubiquitination of factors of the JAK/STAT signaling pathway [26,27]. Viral induction of SOCS expression can be one of the mechanisms of innate immune evasion employed by some viruses [28]. Many regulatory networks of innate responses rely on post-translational modifications (PTMs), such as phosphorylation and conjugation of ubiquitin and ubiquitin-like proteins [29]. For example, the ubiquitination of RIG-I by RIPLET is needed for RIG-I signaling, while the dephosphorylation by DAPK1, an ISG, induced with delayed kinetics, abrogates RIG-I sensing [30,31]. Most signaling molecules involved in antiviral immunity are transcriptionally upregulated early after virus infection [32,33,34]. Paracrine IFN stimulation induces an alert state (also called “IFN primed”) in surrounding cells that allows for a sensitive response to infectious pathogens. Most positive feedback loops appear to be activated early after infection or IFN treatment. Although exceptions may exist, negative regulators tend to be expressed at later time points or become active after long-term stimulation [5,35,36]. These time-dependent differences are essential for antiviral immunity, since negative regulators are active during the early stages of virus infection could compromise the cell’s ability to fight the pathogen. For instance, the ISG DDX6 has been shown to repress the IFN-independent ISG response and consequently promotes the replication of Dengue virus (DENV), Hepatitis C virus (HCV), and HIV-1 [37].

Understanding antiviral processes is further complicated by the activity of viral gene products, which perturb the cellular functions related to the innate antiviral defense and are operative at various levels. Viral strategies can involve inhibition of transcriptional and translational responses, sequestration, or degradation of cellular proteins, mimicking inactive cytokines, or activation of negative feedback mechanisms, to name a few. The intracellular events elicited by virus infections are highly complex and cell-type-, pathogen-, and time-dependent. Many of the above-described functions that are now well-established have been initially identified in excellently performed screening approaches. However, given the described complexity, the experimental approaches to study antiviral immune responses on a large-scale need to be carefully considered and well-adapted to the specific question asked. 

### 1.2. Experimental Systems to Study ISG Responses

In any given screening approach, the chosen cell type has to be carefully considered since cell-type-specific effects are an essential denominator of any experimental design. For screening experiments that involve virus infection, the cell line used should represent a suitable model system, reflecting the natural cellular tropism of the virus. Unfortunately, this is often not possible since many cells that the virus would primarily target are difficult to obtain or cannot be cultured in vitro. The majority of screening approaches make use of tumor-derived cell lines, as they often represent a relatively homogenous population of cells; they are easy to handle and can be grown to high numbers. However, multiple chromosomal aberrations, aneuploidy, and variable chromosome content are widely observed in these cell lines [38]. Moreover, many cell lines bear defects in innate immunity signaling pathways, while the study of innate immunity requires that full or at least partial functionality of this system [39]. An excellent example of this is the cGAS/STING-sensing pathway, which is commonly suppressed in cancers to escape DNA damage responses and sustain genomic instability. Commonly, DNA-sensing pathways are dysfunctional in tumor-derived cell lines [40]. This pathway, however, is not only required to sense DNA viruses but is also involved in the sensing of RNA viruses, most likely through endogenous DNA of nuclear or mitochondrial origin that is released after virus replication [41,42,43]. Therefore, the activity of certain signaling pathways of the cellular model has to be taken into consideration.

Undoubtedly, primary cells would often be better to study innate immunity, but their handling in large numbers is limited by their senescence, and genetic modifications are often challenging to apply. Besides the entire lack of signaling capability, cell lines are—on a quantitative level—often less responsive to the activity of type I IFNs as compared to primary cells, which commonly respond with higher sensitivity (own observations). Thus, the expression profile of ISGs during homeostasis as well as during stimulated conditions could substantially differ when using primary cell lines. Depending on the cell type, the expression of genes involved in the innate immune responses may require activation, since they are dormant under physiological conditions. Such activation steps, however, introduce variables and potential biases in the screening procedure and, thus, need to be considered. In addition, the generation of genetic modifications itself can affect the cellular response. Transfection of reporters, constitutive expression of shRNAs, sgRNAs, and Cas9 can influence the cellular response to infectious pathogens. For example, Cas9 expression has been reported to upregulate p53 signaling, which is well-known to be involved in antiviral immunity [44,45,46]. Off-target effects in CRISPR/Cas9 editing or RNA interference (RNAi) experiments can be minimized during the design stage of sgRNAs or si/shRNAs; however, they cannot be excluded. 

Reporter viruses encoding fluorescently or enzymatically active reporter genes have simplified the abilities to conduct large-scale screens and become a valuable tool. Instead of viruses, replicon systems can also be used to study RNA viruses. Progress in reverse genetics and synthetic genomics facilitates the engineering of recombinant viruses to generate reporter viruses or virus strains with a lower risk assessment. These tools allowed for a quick generation of a fluorophore reporter strain of SARS-CoV-2 during the COVID-19 pandemic [47]. In addition, screening experiments on Ebola virus (EBOV) were made possible by the development of a biologically contained Zaire EBOV strain lacking the transcriptional activator VP30 [48,49].

## 2. Differential Gene Expression Analysis

A powerful tool to identify antiviral proteins is differential gene expression analysis. Since infected organisms are changing their transcriptional profile to respond to pathogens, a plethora of antiviral and immune-regulatory proteins is expressed. Modern transcriptome analysis allows very deep and precise analysis and helps to decipher gene expression patterns indicative of the response to virus infections or cytokines. Hence, it allows the investigators to identify individual genes with virus growth-modulating properties that are differentially expressed after IFN treatment. However, many ISGs show only a mild fold-change in gene expression in the presence of IFN [50]. Gene expression profiles can vary widely, depending on the chosen time point, cell type, and the type of IFN, which makes it challenging to draw a line between ISGs and non-ISGs. Even within the subtype of IFN-α, differences in receptor affinity can lead to substantially different responses [51]. The difference in activity may, in part, be governed by different binding modes that allow IFN subtype-specific conformations on the IFNAR [52]. Consequently, this difference allows IFN subtype-specific gene expression profiles that mediate differences in antiviral resistance [53]. 

Experiments employing single-cell RNA sequencing (scRNA-seq) can further reveal cell-specific gene expression profiles and were already used to identify gene expression profiles linked to the ability of individual patients to control viral gene expression [54,55]. Thus, linking the individual natural susceptibility of cells to the resistance against a given pathogen permits the identification of potentially active genes or at least gene expression profiles. However, a downside to consider is that coverage depth obtained in scRNA-seq is not yet comparable to the depth obtained through bulk sequencing. Therefore, genes with limited expression values can often not be sufficiently detected in single-cell analysis approaches.

While transcriptional analysis is very well-suited to correlate gene expression and gene functions, it is still challenging to deduce functional understanding of individual genes from global gene expression patterns. More mechanistic insights often require loss-of-function experiments (e.g., gene depletion, knockdown, or knockout) combined with stimulation and transcriptional analysis of the resulting gene expression profile. The involvement of a gene-in-gene expression regulation can, for instance, be appreciated in a transcriptional analysis of infected knockout cells. The availability, affordability, precision, and statistical analysis tools that became commonly available substantiated the power of transcriptional analysis to discover interactions between viruses and their hosts.

## 3. Genetic Screens

### 3.1. Gain-of-Function Screens

Gain-of-function screens aim to elucidate gene functions by overexpression and the analysis of the resulting phenotype. Different technologies are presently available that involve using cDNA libraries, lentiviral vectors, or CRISPRa-mediated overexpression (see Appendix A). The power of such screens was demonstrated in fundamental discoveries related to virus sensing. The pattern recognition receptor RIG-I, for instance, has been identified in a screen for genes promoting poly(I:C)-mediated IFN production [56]. Transfection of an extensive cDNA library led to the identification of RIG-I CARD, the active signaling component of RIG-I, which revolutionized our understanding of virus sensing. Similarly, transfection of human and murine cDNAs led to the discovery of STING, as a signaling adapter, involved in the sensing of cytoplasmic DNA and subsequent induction of IFN-α/-β [1]. These studies not only led to the discovery of signaling molecules but also clearly indicated that many ISGs possess direct antiviral activities. This is particularly evident from several comprehensive analyses that tested the virus-specific activity of many ISGs in parallel [57]. The potential antiviral role of over 350 overexpressed ISGs was tested against a broad panel of viruses. Surprisingly, the overexpressed ISGs exhibited both antiviral and proviral effects, indicating that besides expected antiviral functions, many ISGs have regulatory activities in the context of virus infections.

Generally, it appears that overexpression screens are particularly well-suited to identify singly-active proteins that possess the ability to regulate immune responses, while proteins that act in concert with other functional entities are less well-identified. For instance, the most prominent hit with pan-viral antiviral activity was IRF1, a transcription factor responsible for IFN-independent induction of some ISGs. The same screen also identified the DNA-sensor cGAS as a gene with broad antiviral function against DNA-, but also RNA viruses, which may partially be explained by virus-mediated secondary effects such as mitochondrial damage [58,59].

Despite the power of overexpression screens and their contribution to our understanding of antiviral immunity, some points need to be considered. A disadvantage could be the inability to express some ISGs. Toxic effects and overexpression artifacts may cause differential susceptibility or resistance to certain viruses. Besides, the expression of some proteins may interfere with lentiviral particle production used for gene delivery, as those proteins can have anti-retroviral activity [57]. Some proteins may also be susceptible to self-activation when overexpressed. For example, the ectopic overexpression of MAVS, STING, TBK1, and certain IRFs can lead to the activation of an IFN response [60,61]. Even though the obtained data are very valuable, overexpression screens may have an inherent bias towards enzymatically active proteins.

A benefit of protein overexpression screens is that the activity of species-specific isoforms can be easily compared. This is of interest when studying species-specific barriers to virus infections, particularly for emerging viruses or persistent viruses that are cleared in other species. Differences in virus infections between different host organisms can be caused by viral targeting of only some species-specific ISGs. The Rhesus monkey ortholog of TRIM5α (rhTRIM5), for instance, sufficiently blocks replication of human immunodeficiency virus-1 (HIV-1), despite having no effect on simian immunodeficiency virus (SIV) infection [62]. Overexpression of human and macaque ISGs and subsequent infection with different retroviruses identified several antiviral ISGs, some of which were not previously reported to influence HIV-1 infection, e.g., TRIM56 and IDO1 [63]. Similarly, overexpression of a large number of human ISGs was employed to identify factors against Bunyamwera orthobunyavirus (BUNV). This screen revealed the exonuclease ISG20 as the most potent factor that can also restrict other bunyaviruses [64]. Among antiviral ISGs identified by overexpression screens, many exhibited pan-viral activity [57].

While overexpression screens tend to identify PRRs and signaling molecules, proteins that are only active in protein complexes or candidates that need to be expressed in large amounts to exert their antiviral activity appear less prominently enriched in such screens. Examples are IFIT proteins, expressed in high quantities after IFN treatment that act as heterodimeric protein complexes. Expression of individual IFIT proteins exerts only limited antiviral activity compared to the co-expression of different IFIT members [65,66]. The formation of functional complexes can present an obstacle to assess the pro- or antiviral activity of single subunits. In contrast to overexpression screens, functional complexes can be more susceptible to the depletion of single subunits. This effect was demonstrated in the context of antiviral IFIT proteins. Although, it was shown that, among the complex forming units IFIT1, -2, and -3, only IFIT1 directly binds and senses viral 5’ PPP-RNA. However, overexpression of IFIT1 alone did not significantly alter replication of Rift Valley Fever Virus (RVFV), while the depletion of different IFIT proteins influenced virus replication. This indicates that the IFIT complex consisting of several protein family members of which all are important for full antiviral activity, a notion that was backed up by structural analysis of this complex [65,66]. This indicates that complex- and subunit-specific effects can only partially be addressed in functional studies utilizing protein overexpression. 

### 3.2. Loss-of-Function Screens

Although gain-of-function experiments led to the discovery of many important ISGs, factors that do not bear direct antiviral activity often remained inaccessible for these screens. Complementary to overexpressing individual proteins, the depletion of such proteins can cover a broader range of associated functions. In direct comparison, the comparative depletion of target gene products posed technical challenges in the past. Modern technologies made loss-of-function screens versatile and the overall preferred screening strategy to study virus–host interactions. Various techniques were previously utilized to achieve loss-of-function phenotypes, ranging from antibody-mediated inhibition of intra- and extracellular proteins (e.g., cytokines) to genetic knockouts, mediated by homologous recombination. The different available modern techniques vary in their biological performance of depleting a gene product and their technical aspects, such as practicality and cost–benefit ratio.

#### 3.2.1. RNA Interference

Systematic loss-of-function experiments became possible after the discovery of RNA interference (RNAi), enabling the transient depletion of specific messenger RNAs (mRNAs) and their encoded proteins (see Appendix A). While RNAi has revolutionized research by its ease of use, it is being associated with several drawbacks when being used for large-scale screens. Since these experiments needed to be conducted in an arrayed manner, they were highly time- and cost-consuming. RNAi is also limited by a relatively high false discovery rate in genome-wide RNAi screens, requiring secondary testing rounds for top-hit candidates. Furthermore, RNAi is usually not efficient enough to reduce gene expression down to a level equivalent of a null-allele. RNAi instead achieves hypomorphic expression of the target gene [67]. The technical challenges and benefits of RNAi screening approaches were compared by Schuster and Erasimus et al. [68]. Since RNAi does not target the gene itself, knockdowns are not biased by the physical accessibility of the gene locus or the ploidy of the cell. Methodologically, RNAi utilization is a straightforward approach, as the endogenous RNAi machinery is utilized, and no other cofactors have to be delivered. Although it is versatile, RNAi has relatively high off-target effects [69], caused by the RNAi machinery, not requiring full complementarity to the target sequence [70]. Recent developments aim to computationally predict on- and off-target efficiencies to achieve optimal editing efficiency [71,72].

#### 3.2.2. Haploid Screens

Gene trap experiments performed on human haploid cell lines proved to be an effective way to overcome cost and throughput-related issues (see Appendix A). Commonly, haploid screens are used in combination with selection procedures, a function that is often naturally associated with virus infections. Therefore, haploid genetic screens turned out to be elegant screening tools to study host factors that are required for virus replication, particularly genes that are of central importance for virus infection. Foremost, these are entry factors that offer the highest selection benefit. Haploid genetic screens led to the discovery of essential host factors of enteroviruses, Influenza A virus (IAV), RVFV, and Vaccinia virus (VACV), among others, and allowed us to identify the entry receptors of adeno-associated virus (AAV), EBOV, and Lassa virus (LASV) [73,74,75,76,77,78,79].

Haploid screening with a cell line transduced with an ISRE-GFP reporter has been used to identify genes that negatively regulate activation of ISGs expression. By using gene traps, negative regulators of the ISG response, such as the DEAD box RNA helicase DDX6, could also be identified [37]. Although the discovery of CRISPR/Cas9 genome editing pushed haploid genetic screens out of focus, there are still many benefits of this technique. The main one being the random insertion of a gene trap, which can potentially perturb a vast number of genes and noncoding regions, whereas the number of possible CRISPR/Cas9 targets in a genome wide-screen is always limiting and often not targeting less annotated areas of the genome. Overall, genetic screens have a relatively high statistical power. 

#### 3.2.3. CRISPR/Cas9

Another tool to achieve loss-of-function phenotypes is the CRISPR/Cas9-mediated gene editing approach (see Appendix A). The usage of CRISPR/Cas9 results in more robust phenotypes than the transient depletion of mRNA by RNAi. This way, the phenotype of a knockout is commonly more pronounced. Due to the easy implementation into workflows, high ON-target, and relatively low OFF-target effects, CRISPR/Cas9-mediated generation of knockout cells quickly transformed our research and the way loss-of-function experiments and screens are conducted. Computational models nowadays allow for accurate generation of CRISPR/Cas9 target libraries that consider critical parameters such as the transcriptional start site (TSS) and the exon usage in mRNAs [68]. Recent advances also make use of algorithms, which help to predict optimal ON- and OFF-target scores, which is a great asset to interpret screening results [80]. Taken together, CRISPR/Cas9-mediated knockouts are a great alternative to RNAi and can, considering the experimental setting, have numerous advantages.

However, targeting genes essential for cellular viability and the inability to fully predict targeting efficacy still limits CRISPR/Cas9 screens and probably leads to the underestimation of some gene-to-function relationships [81]. Although CRISPR/Cas9-mediated off-target effects are generally considered low, some reports indicate that individual sgRNAs can cause up to 150 double-strand breaks in non-targeted unrelated genetic regions [82,83,84,85].

### 3.3. Validation of Primary Screens

All screening approaches are to some extent error-prone and require downstream validation of the identified candidates. Introduced noise and methodological limitations may result in false-positive identification of targets or over- or underestimation of the observed phenotype. In order to overcome method-specific limitations, an alternative readout should be used to validate the phenotype detected in the primary screening experiment. 

## 4. Assay Design and Screening Format

Besides selecting the type of genetic perturbation and methodological aspects to apply these perturbations, screening approaches can be divided into pooled and arrayed formats, which we discuss in detail below. Both methods have their advantages and disadvantages, with a significant difference being the correlation between genotype and phenotype. In arrayed approaches, cells of different genotypes are physically separated, which makes a direct link to the observed phenotype possible. To the contrary, pooled screens require downstream identification of selected cells. The challenge of all screening approaches is to limit these biases. For simplicity, we here only focus on loss-of-function screens.

### 4.1. Choosing a Target Gene Library

Commonly a library of knockdown or knockout-mediating agents is generated in an arrayed or pooled format. Large-scale screening approaches most commonly employ libraries that target the whole genome. Such pre-assembled libraries are commercially available, and the wide use of such libraries help to establish broadly applicable protocols [86]. However, the parallel targeting of the genome can be complicated by the effects that individual gene depletions have on the cell population. For instance, depleting a protein critically involved in negative regulation of IFN secretion in the given screening system would influence the response of the entire population. Moreover, since the number of genes is large, the statistical power for individual depletions drops. The analysis is thereby complicated and biased in favor of strong phenotypes. The gene selection can also be focused on a functional subset of host genes, for example factors of a particular signaling cascade or a cellular process, e.g., factors that influence virus entry or replication, as well as for factors that directly influence replication, capsid formation, or even virus egress. The focused evaluation of a subset of genes allows us to appreciate more subtle influences of individual gene products. Generally, a more focused library allows for better discrimination of individual pathways and cellular processes.

Such a focused library targeting genes involved in membrane trafficking was used to identify host factors that influence assembly and egress of human cytomegalovirus [39]. In this small screen of 140 targets, identification of host factors involved in late steps of the virus cycle was possible thanks to a two-step readout strategy based on both the fluorescent reporter expression and the amount of progeny virus produced. A large-scale siRNA screen that utilized only a fluorescent reporter readout did not identify those genes [87]. Similarly, van Asten et al. used a focused library targeting secreted proteins. Subsequently, the authors tested the ability of the differently conditioned supernatants to interfere with viral entry [88]. 

### 4.2. The State of the Cell: Requirement of Pre-Stimulation to Allow Appropriate Experimental Conditions

An estimated 10% of all human genes have the potential to be regulated by IFN and are, therefore, considered ISGs [16]. Since these genes are dependent on the stimulus and the duration of stimulation dynamically regulated, screens testing the ability of IFNs to inhibit virus infections requires careful pre-experiments. The expression of many antiviral ISGs is maintained at a low level under non-infected conditions, and only the sensing of an infection or exposure to IFN—either due to paracrine or autocrine feedback loops—elevates their expression levels. Hence, under homeostatic conditions, the expression level of a subset of ISGs might not be substantially different from a knockdown or knockout of this particular gene. Thus, to achieve a pronounced phenotypic difference in depleted conditions compared to controls, pre-treating the cells with IFN might be required to overcome this limitation. However, such additional manipulation steps may differentially bias factors acting during early steps of virus infection and in the induction of IFNs.

### 4.3. Pooled Screens

Pooled screening approaches are the most widely used setup to study loss-of-function phenotypes in vitro. For this, a population of knockdown or knockout cells is generated, commonly achieved by lentiviral-mediated transduction of a library expressing shRNAs or sgRNAs. The transduction of the lentivirus library is carried out at a low multiplicity (MOI < 1) to achieve a single genetic perturbation per cell. Most pooled screens apply selection pressure, and the differences in shRNA or sgRNA sequences before or after selection are evaluated. The correlation between genotype and phenotype can eventually be established using NGS. Either the sequences si/shRNAs or sgRNAs themselves or the edited genes can be utilized as a readout.

The abundance of shRNA or sgRNA that targets genes influencing the selection procedure will change their abundance, quantified by sequencing approaches. As the whole experiment can be carried out in one vessel, this approach can be easily streamlined and applied to a genome-wide scale. 

Pooled screens, however, come with their own set of drawbacks. Already at the step of designing and preparing the library, biases can be introduced. As each target is represented by multiple sh- or sgRNAs, a fractionated loss of the library can introduce significant biases [89]. Moreover, during the experiment, the generated population of knockdown or knockout cells need to be selected, for instance by a challenging virus that induces cell death. These conditions are often overly stringent, as the cells have to survive several rounds of infection before a phenotype can be determined [89]. Similarly, depletion of genes required for cellular homeostasis and replication may lead to adverse effects on cellular fitness and not directly affect the pathogen, complicating the correct interpretation of the resulting data. Although pooled screening approaches increase throughput while limiting costs, certain phenotypes can potentially be overlooked as they could be rescued by surrounding cells. Loss-of-function of factors and receptors involved in auto- or paracrine signaling, for instance in IFN signaling, could appear without a phenotype as secretory signaling molecules are still generated by other cells. Another drawback of this method is the lack of a direct phenotype-to-genotype correlation, thus making downstream identification of selected cells necessary. This can be accomplished by next-generation sequencing (NGS).

The above factors contribute to the relatively low sensitivity of pooled screens, making it challenging to identify weak or intermediate–strong phenotypes. Eventually, the sensitivity of a pooled screen depends on the experimental design. Chou et al. compared seven RNAi-based pooled screens, carried out by different groups, to identify novel host restriction factors of influenza viruses. All screening approaches together identified 1362 potential host factors mediating viral replication of influenza viruses. Only 8% of all hits were found in more than one screening experiment, and no candidates were commonly detected by all experiments [90]. The authors further investigated similarities and variations among the different experimental designs and found that the more similar the experimental setup is, the more common hits were identified. Although the overlap of identified genes between individual RNAi screens is assumed to be low, overlaps of affected pathways and processes can be much higher [91].

Many highly successful genome-wide or focused RNAi screens revealed new regulatory processes of antiviral immunity against Dengue virus (DENV), EBOV, Hepatitis C virus (HCV), HIV-1, Influenza A virus (IAV), Marburg virus (MARV), Sindbis virus (SINV), Vaccinia virus (VACV), West Nile virus (WNV), and ZIKA virus (ZIKV) [92,93,94,95,96,97,98,99,100,101,102,103,104,105,106,107,108,109,110]. In 2015, the first CRISPR/Cas9-mediated pooled knockout screen was designed to study virus–host interactions. Several genes of the endoplasmic reticulum-associated degradation (ERAD) pathway have this way been identified to be essential for WNV infection [111]. In the following years, several novel host restriction factors of IAV and ZIKV have been identified through genome-wide, pooled knockout screens using CRISPR/Cas9 [112,113,114]. Other efforts have been put into deciphering mechanisms of newly emerging viruses such as severe acute respiratory syndrome coronavirus 2 (SARS-CoV-2) and Schmallenberg virus (SBV) [115,116]. In recent years, CRISPR/Cas9-mediated loss-of-function screens increasingly added to our understanding of virus–host interactions of HIV-1, SINV, and different flaviviruses, just to name a few. [117,118,119]. Besides using CRISPR/Cas9-based screening approaches together with a challenging virus, certain triggers of the innate immune system, e.g., endotoxins and cytokines, can also be used to study pathogen–host interactions with a focus on a particular pathway. This way, essential mediators of LPS signaling, IFN-γ-induced cell death, and DNA-damage responses occur during virus infection [120,121,122].

Several novel approaches were also designed to have a more direct correlation between genotype and phenotype and to make the readout more sensitive towards mild phenotypes. OhAinle and Helms et al. designed a novel CRISPR/Cas9-based screening method in which the corresponding sgRNA sequence is packaged into nascent HIV-1 virions [123]. This way, the nascent virions will be enriched for sgRNAs that led to beneficial knockouts for the virus. Paired with an infection system that only allows one round of infection, the authors identified novel host restriction factors of HIV-1. In another approach, Kim et al. aimed to overcome potential artifacts by quantifying the abundance of sgRNA sequences through sequencing mutations in the cellular genome by NGS [124]. In this system, a genome-wide pool of knockout cells was challenged by a picornavirus infection. Instead of reading-out sgRNA sequences, the authors performed whole-genome sequencing of the surviving cells to detect mutations in the target gene directly.

To further address the limitations of pooled screening approaches, methods have been developed that allow easy genotypic identification of cells using in situ hybridization. This way, morphological changes and subcellular organization become available as a readout [125]. Another promising and emerging method is the combination of a pooled genetic screen and the usage of scRNA-seq as a readout [126]. This way, also minor phenotypes can be analyzed.

### 4.4. Arrayed Screens

In an arrayed screen, selection pressure is applied to each cell population containing a single knockdown or knockout, separately. As genotypes are physically distinguishable, a direct phenotype-to-genotype correlation can be established during and after the experiment. Moreover, other readouts, such as the infection kinetics coupled with reporter-viruses and morphological changes, can be monitored by modern life-cell imaging platforms. In comparison to pooled screening approaches, arrayed screens are more laborious and demand more resources. Although the first genome-wide, arrayed CRISPR/Cas9-based libraries were described recently, no genome-wide studies to study virus–host interactions have been successfully conducted yet [127]. Even though less efficient than pooled formats, a tremendous advantage over pooled approaches is the clear separation of genotypes. Thus, depending on the experimental design, making the identification of weak phenotypes achievable. This characteristic was demonstrated by Kim et al. [128]. In this study, the authors conducted a direct comparison between a pooled and an arrayed loss-of-function screen using the CRISPR/Cas9 system. Two pooled screens were performed using the genome-wide GeCKOv2 library or a subset of 1514 target genes. In both screens, only the knockout of coxsackievirus and adenovirus receptor (CXADR)—well-known to be the entry receptor of Coxsackievirus B3 (CVB3)—appeared to have a substantial phenotype. In parallel, the authors conducted an arrayed knockout screen targeting the same 1514 target genes and, among others, identified novel host factors CSDE1 and ACBD3. Disrupting either of these two genes renders the cells resistant to CVB3 infections. This finding demonstrates how pooled knockout approaches might overlook host restriction factors.

## 5. Interaction Screens and Mass-Spectrometry-Based Screens

Gain-of-function and loss-of-function approaches are generally limited as they usually focus on phenotypes associated with one individual target at a time. The complex interactions between viruses and their host cannot be captured as a whole using these approaches. Mass spectrometry, however, paved the way to investigate global changes in host proteomes in response to virus infections. New approaches also made it possible to investigate the regulation of post-transcriptional modification of proteins or specific subsets of the full proteome, such as the secretome or the surface proteome. Every dataset adds to the understanding of virus–host interactions and advances virus research.

### 5.1. Interaction Screens

Temporary or permanent physical interactions convey most virus-mediated alterations of the host cell. Identifying virus-targeted proteins is of great interest and a first step to deciphering viral pathogenesis in order to understand downstream effects. Among the first approaches to detect virus–host interactions based on ectopic protein–protein interactions, yeast two-hybrid assays (Y2H) were used [129]. Further developments of this method allowed for the discovery of numerous interactions between proteins. Among interactions between viral proteins of HCV, different herpesviruses, and VACV [130,131,132,133,134], as well as of virus–host interactions of EBV, HCV, IAV, SARS-CoV-1, VACV, and VSV [135,136,137,138,139,140]. However, Y2H assays have some limitations, as they only allow the study of direct interactions between two proteins. Moreover, since Y2H screens are based on the activation of a transcriptional transactivator, these interactions have to take place in the nucleus. This, however, is often not the case and can lead to a high false-negative as well as false-positive discovery rate. Besides these issues, complex-forming proteins with indirect interactions cannot be investigated. Despite the drawbacks of Y2H screenings, a positive evaluation typically suggests direct interactions. In this regard, Y2H can help to de-convolute protein–protein interactions within a protein complex. Parallel advances in mass spectrometry made it possible to use affinity-purification, followed by mass spectrometry (AP-MS), to overcome these limitations.

### 5.2. Proteomic Approaches

In the past years, the identification of host proteins interacting with viral structures, e.g., proteins or nucleic acids, has fueled the understanding of viral pathogenesis. Proteomics has thus become a standard tool to study virus–host interactions. Even though proteomics is elaborative and single precipitations have to be investigated, it makes it possible to study the formation of protein complexes in native conditions. It also helps to unveil how protein complexes dynamically change after virus infection or IFN treatment, therefore giving unique and unbiased insights. In addition, in a non-infected state, studying the interaction partners of ISGs, and thus their functional assignment to cellular processes, aids our understanding of the innate immune system [141]. However, proteomics approaches require sophisticated statistical analyses as well as careful experimental design. We want to refer to excellent reviews that, in-depth, describe proteomics approaches in virus-infected and non-viral settings [142,143,144,145].

### 5.3. Virus as Baits

To study the interaction of viral proteins with the host proteome, individual tagged viral proteins can be precipitated to determine host interaction partners. Although this approach does not allow us to study the viral protein in the context of viral infection, it allows us to gain fundamental knowledge on the activity of individual viral proteins. Andrejeva et al., for instance, used tagged V proteins of different paramyxoviruses, which are known pathogenicity factors, as baits to co-precipitated binding partners, followed by MS analysis. The main interactor of V proteins was identified to be melanoma differentiation-associated protein 5 (MDA5). Several years later, it became evident that MDA5 belongs to the family of RIG-like receptors and has an essential role as a PRR [146]. This approach demonstrated that viral proteins can be used as baits to discover signaling molecules relevant for innate immunity. This concept was taken further to systematically study the interactome of viral immune-regulators. It showed that these proteins, though derived from different viruses and virus classes, evolved to target a conserved network of proteins [147]. AP-MS was also performed to unveil virus–host interactions by tagging all viral proteins of HIV-1, EBOV, ZIKV, and SARS-CoV-2 [148,149,150,151]. This approach allows us to identify connections between viral proteins and proteins of the innate immune system, supporting the notion that viruses have to evolve sophisticated and broad strategies to escape innate immune surveillance. Besides using viral proteins as baits, viral nucleic acids can also be used to identify cellular proteins interacting with viral nucleic acids. Such proteins can reveal PRRs as well as restriction factors. For instance, this led to the discovery of IFN-induced protein with tetratricopeptide repeats 1 (IFIT1) as a novel nucleic-acid-binding protein and IFI16 as a protein that is involved in the induction of type-I IFNs [65,152].

### 5.4. Secretomics

Besides the global changes on the host proteome in response to virus infection, certain subsets of the proteome, such as the secretome or the surface proteome, can undergo substantial changes. By studying those changes, the mechanisms of cell-to-cell communication, mediated by soluble factors such as chemokines and cytokines can be studied, adding to a deeper understanding of virus–host interactions. Meissner et al. and Koppenol-Raab et al. analyzed TLR-stimulation signaling cascades and the secretome of macrophages after stimulation with different TLR ligands and then mapped their differential regulations in a time-dependent manner [153,154]. These analyses revealed several hundred proteins differentially present in the supernatant of cells exposed to TLR ligands, which may allow very intense crosstalk between cells. A main issue in secretomics analysis is the identification of trace amounts of cytokines, covered by highly abundant proteins. Although the dynamic range of mass spectrometry analysis allows highly sensitive identification, cytokines are at the lowest level of expression. Recent technological advancements in MS, as well as biochemical enrichment strategies, allow for the identification of secreted proteins. These could be secreted proteins with signaling capacity (e.g., cytokines) or molecules that are only released upon certain cellular conditions such as cell stress (e.g., danger-associated molecular patterns—DAMPs). The distinction between actively secreted and passively released proteins is a main challenge in secretome analysis. In part, this can be overcome by elegant metabolic labeling approaches that allow us to enrich newly synthesized proteins. However, cytokines that are pre-formed in cells and are released upon a maturation step (for instance, IL-1b, IL-18) cannot easily be discriminated from factors that are released upon cell damage. Secretome analysis holds the promise of fundamental discoveries in our understanding of cell-to-cell communication, but careful analysis and additional functional experiments are required to fulfill this expectation. 

## 6. Future Perspectives

**Figure 2:**
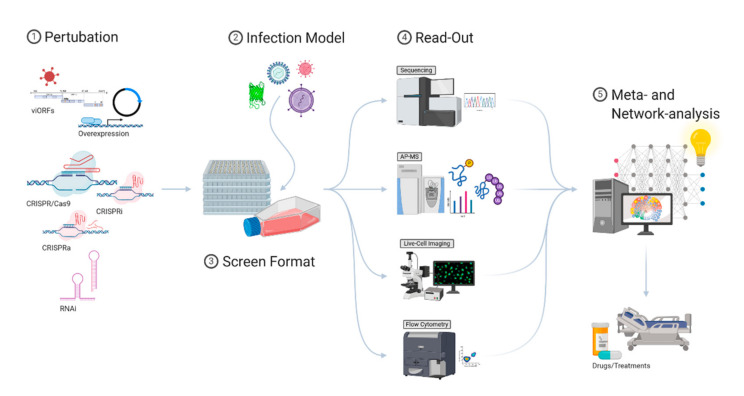
An extensive portfolio of genetic perturbation approaches (1) can be used to modify the protein expression levels to study the contextual function of specific gene products. With a suitable infection model system (2) (e.g., reporter viruses) and a fitting screening format (3), a large number of target genes can be studied on a large scale. Several different methods can then be used as a readout for gene/protein functionality (4). Different datasets can then be merged and fed into meta- and network-analysis algorithms (5). Gathering detailed insights from different experimental settings, a global picture of the innate immunity landscape can be drawn. It is critical to understand virus–host interactions in detail to develop novel antiviral drugs and treatments at the time they are needed.

The innate immune system has evolved, driven by co-evolving pathogens, into a highly-structured and complex multi-factor defense barrier. The sophisticated arrangement of obstructions makes it challenging for a pathogen to adapt to its host and overcome the innate immune response. At the same time, the complexity of the innate immune system is challenging to comprehend and explore therapeutically. In the past, the IFN-induced antiviral response has mostly been studied by individual perturbations of cells. This way, many of the currently known viral restriction factors have been identified. However, these approaches cannot address the extensive interplay of virus and host and often do not allow us to study connections between different pathways. Such connections can best be studied by large-scale screening methods. Although promising, it is still challenging to identify and functionally verify those connections. Modern screening approaches require reliable readouts, in-depth knowledge of virus-affected pathways, and computational methods to integrate diverse datasets (Figure 2). Considering contributing, synergistic, and antagonistic effects, the antiviral landscape of the innate immune system can be understood on a systems level, and drug-based interventions can be successfully identified. While major advances have been made in the last few years, the integration and interpretation of highly complex datasets currently remain one of the main bottlenecks that need to be addressed in the future.

## Appendix A. Box 1 (Methodology)

RNA Interference (RNAi).

After discovering the suppressive effect of small interfering RNAs (siRNA) on the abundance of mRNA transcripts, RNAi was widely utilized as a screening method for genome-wide studies [155]. RNAi uses the canonical pathway for microRNA (miRNA) processing to generate short, interfering pieces of single-stranded RNA (ssRNA) that are processed by Dicer enzymes functioning as endoribonucleases. siRNA duplexes are then loaded onto RNA-induced silencing complexes (RISC) by a RISC loading complex (RLC). Active RISC is subsequently bound to its target region on mRNA molecules. The associated ribonuclease Argonaute then initiates the degradation of the target mRNA [156].

Haploid Genetic Screens.

One of the approaches to loss-of-function screens is mutagenizing cells with integrating retroviral gene trap vectors that contain a splice acceptor and disrupt the expression of the gene they integrate into. Gene trapping screens in mammalian cells have been greatly facilitated by the use of a haploid cell line in which the disruption of only one allele is needed to target endogenous protein expression [77]. The nearly haploid adherent fibroblast cell line HAP1 was derived from the chronic myelogenous leukemia (CML) KBM-7 cell line, which was additionally transduced with retroviral vectors encoding Yamanka factors in attempts to induce pluripotency [74]. More recent genetic approaches employing CRISPR/Cas9 mediated deletion were used to entirely remove a diploid region of chromosome 15 and create a fully haploid cell line (Ehap—engineered HAPloid) [157]. Haploid screening has been the first approach that allowed for unbiased genome-wide screening and led to important discoveries, especially in the study of virus entry.

CRISPR/Cas9.

Clustered regularly interspaced short palindromic repeats (CRISPR)/CRISPR-associated (Cas) are the central entity of an antiviral defense system in bacteria [158]. This RNA-guided endonuclease system can introduce mutations in a targeted manner and is now widely used for genetic engineering. After its discovery, the system was optimized to generate a single-guide RNA complex (sgRNA) consisting of a target-specific crRNA and a transactivating RNA (tracrRNA). Cas9 recognizes this sgRNA complex and is guided to the specific target locus by the 20 nucleotide long crRNA, where a DNA double-strand break (DSB) is then induced [159]. A DSB causes immediate activation of repair pathways; in the case of CRISPR/Cas9-mediated DSBs, there is a combination of microhomology-mediated end joining (MHEJ) and non-homologous end-joining (NHEJ) pathways, which results in an error-prone repair [160]. During the repair, bases might be randomly inserted or deleted, potentially leading to a perturbed nucleotide sequence. This can lead to a shift in the reading frame, which could potentially generate a premature termination codon (PTC) and make the transcribed mRNA susceptible to nonsense-mediated decay (NMD), and thus causing a substantial reduction on the transcript level [161].

CRISPRa/i.

Technological advancement made it possible to use CRISPR to sterically inhibit transcription using CRISPR interference (CRISPRi) and to induce expression by CRISPR activation (CRISPRa). CRISPRi utilizes an engineered mutant of Cas9—dead Cas9 (dCas9)—without endonuclease activity, rendering it unable to induce DSBs. Guided dCas9 blocks the genetic locus from transcription by either interfering with the binding of RNA polymerase or other transcription factors or direct polymerase stalling [162]. Although few screens have been conducted in the context of antiviral immunity using this new platform, it is likely that these additional tools will further aid our understanding of antiviral immunity.
